# Usage of copper nanoparticles as a nematicidal agent for root-knot nematodes in naturally infested open-field pepper

**DOI:** 10.1186/s13568-025-01964-9

**Published:** 2025-11-06

**Authors:** Rehab Y. Ghareeb, Marwa Muhammad Abu-Serie, Huda Salem Alrawiq, Ali Hamdy, Sanaa S. A. Kabeil, Sahar A. Zaki

**Affiliations:** 1 Plant Protection and Biomolecular Diagnosis Department, Arid Lands Cultivation Research Institute (ALCRI), City of Scientific Research and Technological Applications (SRTA-City), New Borg El Arab, Alexandria 21934 Egypt; 2 Medical Biotechnology Department, Genetic Engineering and Biotechnology Research Institute, (GEBRI), City of Scientific Research and Technological Applications (SRTA-City), New Borg El‑Arab, Alexandria 21934 Egypt; 3 Botany Department, Faculty of Science, Sebha University, Sebha, Libya; 4 Environmental Biotechnology Department, Genetic Engineering and Biotechnology Research Institute (GEBRI), City of Scientific Research and Technological Applications (SRTA-City), New Borg El-Arab, Alexandria 21934 Egypt; 5 Department of Protein Research, Genetic Engineering and Biotechnology Research Institute, City of Scientific Research and Technological Applications (SRTA-City), New Borg El-Arab, Alexandria Egypt

**Keywords:** CuONPs, *Meloidogyne incognita*, Plant protection, Growth promoting, Cytotoxicity assay, Nematicide, Nematode management

## Abstract

Root-knot nematodes (*Meloidogyne* spp.) cause significant economic losses and have a profound impact on the agricultural sector, while commercial chemical nematicides have been found to cause pollution and are harmful to human health. The unique qualities and possible advantages of metal-based nanoparticles have gained considerable interest in plant disease control. This study synthesized, described, and evaluated CuONPs against the *M. incognita* nematode in terms of female biological cycle arrest, reduction of egg hatchability, and second juvenile mortality in pepper plants. It was done in both in vitro and open-field trials. Copper nanoparticles tested concurrently in graded concentrations (100, 75, 50, 25, and 12.5%) were evaluated. CuO nanoparticles were revealed to be effective nematode agents in the in vitro assay against eggs and second juvenile stages (J2s) of *M. incognita*, with a 100% concentration after 48 h of exposure, causing 87.9% larval mortality and 57.5% egg hatchability reduction. The open field experiment on pepper CuONPs application in direct irrigation showed 100% results with a concentration of 100%, an 84.4% decrease in egg masses/plant, a 92% decrease in J2s/100 g soil, and ultimately, an effect on the female's life cycle with an 83% decrease in number and an 89.6% reduction in galls/plant. Furthermore, pepper plants treated with CuONPs showed fresh and dry weights for the shoots and roots in open field trials. Finally, the safety effect of CuONPs on the growth of normal human cells was evidenced by its higher safety on normal human lung and skin cell lines (WI-38 and HFB4, respectively). CuONPs exhibited significantly effective growth parameters and strong nematicidal effects against nematodes, so they could be considered a safe and potential nanofertilizer and nematicide agent.

**Graphical abstract:**

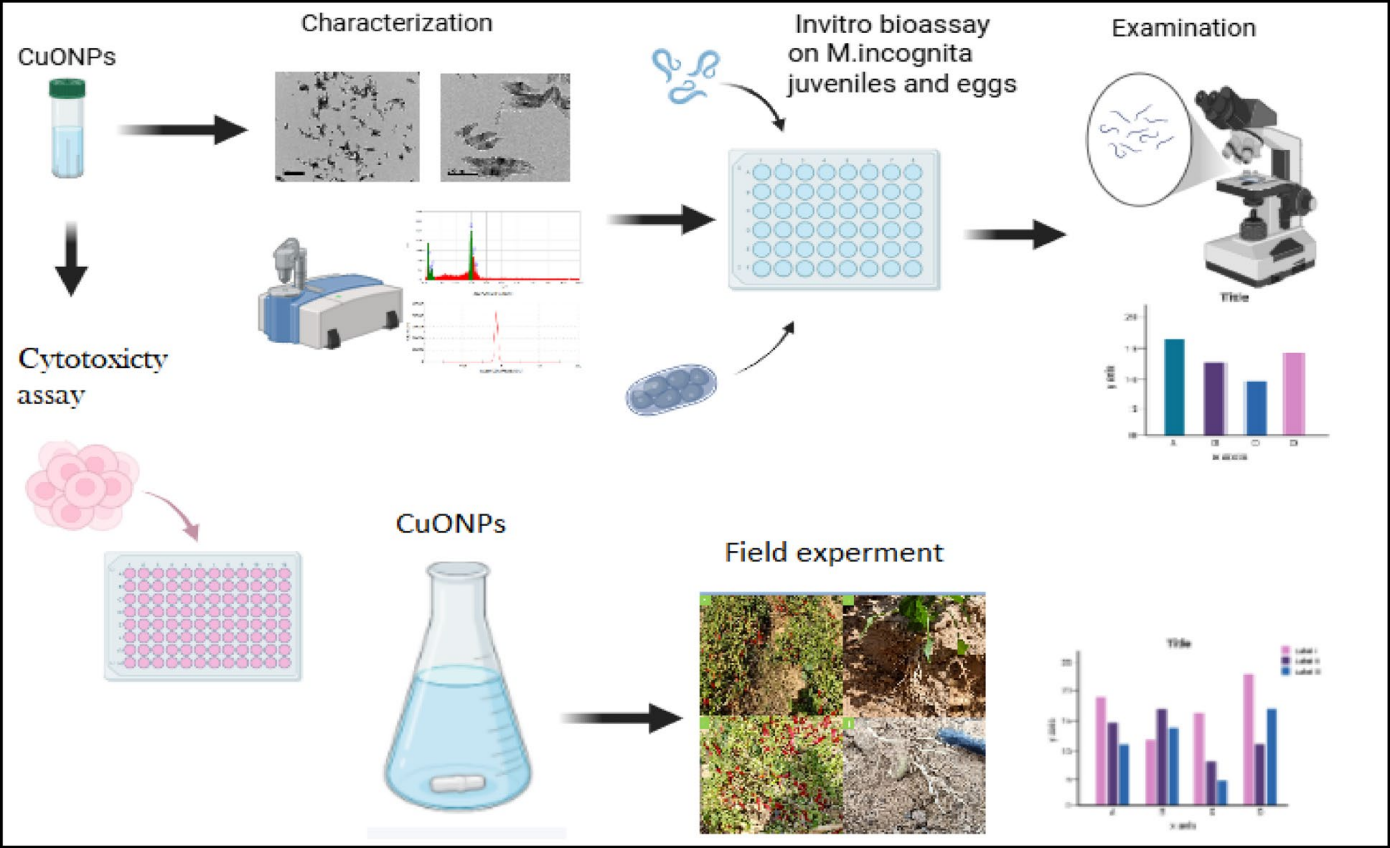

The importance of incorporating copper nanoparticles as a nematicidal particulate to improve the growth and yield of pepper plants, along with the fertility and health of the soil.

## Introduction

Peppers belong to the Solanaceae family, which includes other important vegetables such as tomatoes, potatoes and aubergines. All types of sweet and hot peppers include chili peppers and red pepper (Zayed et al. 2017). Pepper is an important vegetable crop in the world and in Egypt.

However, they are often attacked and damaged by many PPNs, especially root-knot nematodes in Egypt. As time goes on, the nematode population and grows reproduces rapidly, and the leaf disease increases. Abd-Elgawad (2020) reported that the annual loss of pepper due to damage caused by plant pathogenic nematode (PPN) in Egypt is 22% of the total yield, which was 183,489.6 tons of yield loss per year in 2012. However, the effect of PPN populations on pepper crops varies depending on the toxicity and level of associated nematode species, as well factors that interact with cultivated pepper (Abd-Elgawad 2016a; b).

*Meloidogyne* spp., commonly known as root-knot nematodes, are notorious plant parasitic nematodes that cause significant crop losses in the production of fruits and vegetables (Singh et al. 2015; Gareeb et al. 2019; Alfy et al. 2025). Plant-parasitic nematodes, especially *Meloidogyne* spp., pose a significant threat to the majority of agricultural crops in Egypt, particularly those with root systems (Ibrahim et al. 2014).

These agricultural pests have been traditionally controlled using synthetic organic chemical nematicides (Ntalli et al. 2011). However, due to environmental concerns, many of these nematicides such as synthetic carbamates, organophosphates, and organohalide pesticides have been banned recently (Council Directive 91/414/EEC, Regulation 2009/1107/EC.OL and Directive 2009/128/EC). As a result, there is an urgent need to develop alternative nematode control measures and green nematicidal products that are less toxic to non-target organisms, the environment, and humans (Chitwood et al. 2002; Khan et al. 2023). The chemical pesticides of nematodes are typically more effective than other methods, but their use is limited due to their harmful effects on humans and the environment. As a result, farmers are becoming less interested in chemical nematodes as their harmful impact becomes more widely known (Kausar 2021).

Nano-agriculture involves the use of nanomaterials, such as nanopesticides and nanofertilizers, to protect crops and improve plant health in a sustainable manner (Marschner et al. 2007; Bansal et al. 2025). Recently, methods based on nanotechnology have been used to control plant pathogens because of their unique characteristics, such as their small size, large surface area, higher chemical reactivity, and electric conductivity. Metal-based nanoparticles (NPs) have gained attention due to their unique physicochemical properties and biological activities (Ghareeb et al. 2022). The impact of metal-based NPs on plant growth and their accumulation in food sources is dependent on the applied dose and varies among species. The effectiveness of metal-based NPs is influenced by factors such as the characteristics of the nanomaterial and environmental conditions. The 4s' parameters (size, shape, surface chemistry, and structure) of NPs can significantly affect their biological properties, while their environmental behavior (mobility, reactivity, toxicity) must be fully understood. Therefore, it is necessary to explore the effects of metal-based NP formulations on different soils and crop plants to expand sustainable agricultural production for higher yields and safer products (Yruela 2009; Verma et al. 2022).

Cu is one of the crucial trace elements necessary for maintaining the systematic growth and evolution of plants. As, it is the vital axis of many enzymes closely intricate in various biotic activities such as, the manner the transportation of proteins, metabolism of a cell wall, respiration/electron of photosynthesis transfer, Deoxyribonucleic acid (DNA) synthesis, oxidative stress and the signals of hormone transduction (Gong et al. 2021). Notably, Cu insufficiency leads to the decrease of plant growth, distortion and chlorosis of young leaves, crimping of the leaf edge, destruction of the apical meristems, and decline in seed setting average (Epstein and Bloom 2005; Chen et al. 2022a, b).. Because Cu deficiency reduces the formation of cell walls and lignification in nearly all tissues, including the xylem, its secondary effect may not be the transport of water (Burkhead et al. 2009; Ishka and Vatamaniuk 2020).

A Cu shortage has an acute smack on pollen and the development of embryos, seed and pollen viabilities, and the final yield of seed and fruits. Cu-correlated proteins are crucial in the transportation of electrons in the chain of chloroplasts and mitochondria. Besides, Cu associates in the reactions of photosynthetic PSII (responsible for the light-driven oxidation of water, producing oxygen, protons, and electrons) is self-reliant on plastocyanin and stimulates the oxygen-change activities in vitro. Chen et al. (2022a, b). Copper is essential for the activity of Cu/Zn-SOD, the enzyme that interacts with ethylene, laccase, polyphenol oxidase, and other enzymes that require multiple copper ions. Certain copper oxidase enzymes, like amine oxidase, which interacts with the cell wall, play a role in converting the polyamine putrescine (1,4-diaminobutane) to hydrogen peroxide (H_2_O_2_) (Festa and Thiele 2011). This conversion is crucial for the lignification process, the cross-linking of cell wall proteins, and programmed cell death. Hydrogen peroxide acts as a signaling molecule, participating in various physiological and biochemical functions. It can influence plant growth and development, boost plant resistance to stress, reinforce cell walls, enhance photosynthesis, slow down the aging process, and improve the movement of stomata (Chen et al. 2022a, b). NPs have significant effects on metabolic and physiological functions. By enhancing defense genes, ionic homeostasis, anti-oxidant activities, and membrane stability, CuONPs control plant tolerance to various stressors and prevent plant diseases (Noohpisheh et al. 2020; Chattha et al. 2022; Zhou 2025). Pesticide formulations based on copper ions (Cu⁺ and Cu^2^⁺) and reactive oxygen species (ROS), which are the true "active forms" or biocidal agents released by the nanoparticles, have been used for a long time to protect crops. Because copper in nanodimensions produces a significantly higher active form of copper at a significantly slower rate, the metallic copper nanoparticle itself is not the primary weapon, but rather a delivery system that releases these potent active forms in a controlled and highly efficient mannercopper nanoparticles and their composites may be able to control a variety of plant diseases. The growth of *Capsicum annuum* plants depends on copper and iron because they are essential for photosynthesis and other cellular physiological processes, and their absence could cause structural damage (Kausar 2021). Additionally, CuONPs have been used in the development of fertilizers, growth modulators, pesticides, herbicides, and soil remediation. Copper formulations can also act as antagonists against plant diseases, making them helpful in formulations. Copper-based nanoparticles (NPs) have considerable potential in agriculture due to their bio-affinity and low toxicity. Moreover, copper nanoparticles and their nanocomposites have effectively managed plant diseases and generated a far more active form of copper. Additionally, the inhibition efficiency rises in proportion to the concentration of CuONPs (Van-Viet et al. 2016). Copper nanoparticles are effective against a variety of microbial pathogens in studies on their bioactivities. Likewise, it has been reported to have anti-parasitic and anti-cancer properties. Since the salts of metallic copper are lesser than those of silver, the overall technology is cost-effective, and thus, copper nanoparticles can be used as one of the inexpensive alternatives to silver nanoparticles (Ingle et al. 2014). Currently, scientists are exploring the realm of nanotechnology, which offers numerous uses in agriculture and biomedical research, including the discovery of novel nematicides and anthelmintic medications that are less harmful to both the environment and the host. Studies are being conducted with metal nanoparticles as potential nematicides for fighting plant-parasitic nematodes to enhance crop yield. Recently, it was discovered that copper nanoparticles are both non-toxic and environmentally sustainable, leading to the development of new, effective and better nematicides. Interesting characteristics of copper oxide nanoparticles (CuONPs) include their high effectiveness in killing bacteria, accelerating reactions, inhibiting disease cells, and serving as surface coatings, among other uses. Additionally, natural CuONPs are plentiful and affordable. These nanoparticles, which possess notable physicochemical properties, occur in various oxidation states, such as Cu0, CuI, CuII, and CuIII. They are thermally stable, chemically inert, and their approximately 2 eV band gap energy gives them significant relevance. Compared to organic materials, CuONPs have a longer shelf life because they are inorganic.

The goal of this study was to investigate whether copper nanoparticles could be used to fight plant parasitic nematodes as a type of nematicide for peppers, emphasizing the importance of copper nanoparticles with low toxicity in an ecosystem against root-knot nematodes (*Meloidogyne incognita*) through experiments in the lab and in open fields.

## Materials and methods

### Copper oxide nanoparticles preparation

A standard procedure was followed to synthesize CuONPs via the chemical precipitation method (Pandey et al.^2012^; Rangel et al. 2020) with slight modifications. To perform the chemical synthesis of CuONPs, 5.4 g of sodium hydroxide pellet and 9.0 g of copper (II) chloride dihydrate were dissolved in ethanol separately.

The amount of ethanol used is as minimal as essential for copper (II) chloride dihydrate and sodium hydroxide (Sigma-Aldrich) to dissolve independently. The sodium hydroxide solution was continuously stirred at room temperature while a drop wise addition of copper (II) chloride dihydrate solution was made. As the reaction progressed, the solution's color changed from green to bluish green and then to black, and the black precipitate was copper hydroxide. After the black precipitate was separated using a cold centrifuge at 6000 rpm for 30 min, the sodium chloride salt solution was rinsed out using ethanol and deionized water. The precipitate was then dried in a dryer set at roughly 70 °C for a day. To produce crystalline CuONPs, the dried sample was annealed at different temperatures, to obtain the powdered nanoparticles; the annealed sample was subsequently ground. The CuONPs were characterized using the produced powder sample and stored in tubes for study. Five different concentrations (100, 75, 50, 25, and 12.5%) of Cu nanoparticles were prepared for use in this study. Suspensions of these concentrations were applied to J2s and eggs of root-knot nematodes (*M. incognita*) in cell line *plates* (9 wells). The plates were divided into three replicates and treated with a commercial nematicide or sterile water as a control. All plates were incubated at 27 ± 2 °C, and egg hatchability and J2s mortality were recorded after 12, 24, and 48 h from exposure time The juveniles were considered dead if the nematodes seemed still in the plain water, and the experiment was repeated twice.

### Characterization of the synthesized CuONPs

#### Ultraviolet visible spectrophotometer

The UV–Vis spectrophotometer was used to measure the UV–visible absorption of CuONPs at room temperature. A T60 Visible Spectrophotometer (PG Instruments Limited) was used to check how many light copper nanoparticles absorbed at a wavelength of 580 nm. Centrifugation at 8,000 × g for 15 min was used to collect the dark blue solid product, which was then carefully cleaned twice with fresh distilled water and absolute ethanol. The final product was baked overnight at 35 °C. For additional analysis, the dried sample was ground into a powder and kept in drops of absolute ethanol.

#### Scanning electron microscopy

The alteration in morphology and color of the CuONPs was noted using a scanning electron microscope (SEM). The chemically synthesized CuONPs, which reap at 8.000 g for 20 min at a cool temperature of 4 °C, are swept by absolute ethanol and firmed by 2% glutaraldehyde and 1% osmium tetroxide (OsO4). After firming, the CuONPs samples are washed, escorted with ethanol, and desiccated in rising concentrations of ethanol (50, 75, and 100%). The dried CuONPs were then covered with a fine coating of gold. The particle size of CuONPs was detected by scaling the size of randomly picked particles in samples with the SMILE VIEW software and a JEOL JSM-6490 (JEOL, USA).

#### Transmission electron microscopy and Energy dispersive X-ray absorption

TEM is used to scan a finely focused electron beam across the surface of a specimen. The reflected signals are collected, and their intensities are displayed on a cathode-ray-tube screen by brightness modulation. As already indicated, the method allows specimen magnifications to 300,000X while maintaining a large depth of focus. The ease of sample scanning by scanning electron microscopes over large distances is quite appealing, in that a large sample viewing area is first surveyed (at generally low magnification) to seek particular areas of interest, followed by high magnification of those specific areas for subsequent detailed investigations. The TEM is also extensively employed for the generation of dimensional and spatial relationship details of structure elements. A tiny amount of the dry sample is scattered in DH_2_O to create TEM CuONP samples, which are then inclined by placing a few drops of the mixture on a copper grid (Field Emission Transmission Electron Microscope, JEOL-JEM-2100F).

#### Fourier transform infrared spectroscopy and X-ray diffraction

The practical biomolecules in the CuONPs samples that the CuONPs formation could control were explored and characterized by an FT-IR spectrometer (FTIR-8400S, Shimadzu, Japan). To detect the constitution of the dried CuONPs, they are pressed into a lessened little piece with potassium bromide (KBr) fine powder and scrutinized at wavelengths ranging from 400 to 4000 nm using powder of KBr. The crystal structure of CuONPs was determined using X-ray powder diffraction (XRD-7000 model, Shimadzu, Japan) with CuKα radiation (λ = 1.54060 Å) and a gradual scanning method (2θ range from 5° to 80°) at a speed of 4θ/min. The midpoint crystal size (D) was recorded (Salama et al. 2023), Using Debye–Scherrer's equation, the full width at half-maximum of the diffraction peak was used to determine the average crystal size

$$ D = \frac{0.9\lambda }{{B_{2\theta } \cos \theta_{\max } }} $$

where B2θ is the angular width in radians at an intensity equal to half of the maximum peak intensity, θ is the diffraction angle, λ is the characteristic wavelength of the X-ray used (1.54060 Å), and D is the average crystallite size in Å.

### Zeta analyzer 

As before, the average size and stability of CuONPs were assessed at room temperature and neutral pH. To assess the average size distribution, 100 μg/ml of each sample was dissolved in ethanol and subjected to a 5 min sonication. The zeta potential and size of the CuONPs were then measured using the light dynamic scattering method using an electrophoretic light scattering device (Malvern Zetasizer nanosizer, at CRTA, City of Alexandria, Egypt).

### Root-knot nematode (Meloidogyne incognita) SOURCE

Nematode inoculum was produced using pure cultures of *M. incognita* on peppers (*Capsicum annuum)* grown in the greenhouse of the City of Scientific Research and Technological Application in Alexandria. Under a stereoscopic microscope (LABOMED; Labo America, Inc. USA), three months after the infestation of the pepper plant with *Meloidogyne* sp., adult females of root-knot nematode were collected from galled roots for species identification by the characteristics of the perineal pattern technique (Holbrook 1983). Plants were uprooted and egg masses were collected from the galls of infected roots with a needle. The roots were cut into small pieces (2–3 cm) and then placed in a flask containing 0.5% NaOCl solution described by (Hussey et al. 1973).

Active juveniles (J2S) of nematodes were obtained by using the Baermann plate technique (Ayoub et al. 1980). The egg masses in the galls were harvested and picked up with a needle, and the retrieved galled roots were cleaned with tap water to eliminate any remaining soil particles. To encourage hatching, egg masses were cultured in *petri* plates with distilled water for 48 h at room temperature (27 ± 2 °C). When J2s hatched, active samples were gathered.

### In vitro assessment of CuONPs on juvenile mortality of M. incognita

To evaluate the potency of CuONPs solution on juveniles (J2s) of *M. incognita,* the mortality percentages were inspected with different concentrations 12.5, 25, 50, and 75, to 100% compared with the control treatment under lab experiments. The bioassay was conducted in cell line *plat*e with three replicates of each treatment, comprising roughly fifty newly hatched second-stage juveniles (J2s) per replica compared to the control treatment dH_2_O, and the experiment was repeated twice. At 24, and 48 h, the juvenile mortality was measured on the plates containing the J2s with CuONPs, which were cultured at 27 ± 2 °C. After treatment, juveniles were transferred to plain water. Larvae were considered alive if they moved in a curved pattern, but they were considered dead if they did not retrieve movement after being transferred to water and when examined with a fine needle (Ghareeb et al. 2022; Mohammad et al. 2022) the experiment repeat twice. The formula used to determine the percentage of juvenile mortality was as follows (Kamalanathan et al. 2023)

$$\left( {\text{M}} \right){\text{Mortality}}\% = \left( {\frac{\begin{gathered} {\text{Total number of J2s in control}} \hfill \\ - {\text{Number of alive J2s in treatment}} \hfill \\ \end{gathered} }{{{\text{Number of total control J2s live}}}}} \right) \times 100$$

### In vitro assessment of CuONPs on egg hatchability of M. incognita

The bioassay to determine the hatchability of *M. incognita* eggs was performed using a sterile 6-well cell line culture *plate* (SPL Life Sciences Co., Ltd., Korea), containing approximately 100 eggs in each well. Five concentrations (12.5, 25, 50, and 75, to 100%) of the CuONPs were evaluated, as well as dH_2_O and Oxamyle serving as controls. The plates containing dH_2_O served as controls.

Every experiment was done twice, and five duplicates were created. After 3, 7, and 15 h of incubation at 27 ± 2 °C, the hatching of each treatment plate was noted following each count, and the eggs were moved to new plates containing the same concentration of particles after being cleaned in 1 mL of dH_2_O in their plates. In accordance with this, the percentage of inhibition of egg hatch was computed.

$$ \% {\text{Egg hatchability = }}\frac{{{\text{Total egg in control}} - {\text{Egg in treatment}}}}{{{\text{Egg}}\;{\text{hatching}}\;{\text{in}}\;{\text{control}}}} \times 100 $$

### Effects of CuONP on pepper plants under open field conditions in naturally infested soil with root-knot nematode

The experiments of field were conducted in Nubaria, El-Bohira Governorate, Egypt (30° 40′ N 30° 04′ E/30.667° N 30.067° E) in May 2022 in a field that was naturally heavily infested with *M. incognita*. The soil there is sandy clay loam with low organic matter, very high calcium carbonate, and no salinity. Macro-elements like nitrogen were moderately available at 23.2, while phosphorus and potassium were 21.9 and 123.1, respectively. For micronutrients, Fe, Cu, and Mn were present at 3.7, 0.9, and 3.5, respectively, whereas Zn was 0.35, and B was present at 1. At the start of the study, temperatures were generally high, with monthly averages ranging from 24 to 32 °C. Relative humidity was slightly below the long-term average, ranging between 65.5 and 68%. Forty-five-day-old pepper (cv. Gold) seedlings were implanted in the field infested by the nematode. With 6 treatments and 20 plants per treatment, the experiment was set up in a completely randomized block design, as 50 ml of each concentration of CuONPs were applied as a drench before transplanting, and the experiment was repeated twice. Following the extraction of the final populations of *M. incognita* nematodes from the soil, the reduction percentage of J2s was calculated by counting the number of J2s/250 cm^3^ of soil, galls/root system, egg masses/root system, and reduction % of eggs/root system. Lastly, by calculating the maximum plant, shoot, fresh, and dry weight, the increase in plant growth parameters was assessed.

### Detection of synthesized CuONPs cytotoxicity in vitro

The toxicity of the studied CuONPs was tested using human lung fibroblast cells (WI-38, passage number 31) and HBF4 at the Medical Biotechnology Department, Genetic Engineering and Biotechnology Research Institute, City of Scientific Research and Technological Applications (SRTA-City), Egypt. These cell lines were obtained from the American-Type Culture Collection (ATCC). After culturing cells in DMEM media containing 10% fetal bovine serum and seeding in sterile 96-well culture plates (1 × 104 cells/well), cells were incubated in a CO2 incubator (37 °C) for 24 h. Following cell attachment, serial concentrations of CuONPs were incubated with Wi-38 or HBF4 cells for 72 h following 24 h of cell attachment. MTT was used to measure the viability of the cells according to the Mossmann method (Gareeb et al. 2019). Briefly, MTT (Sigma, USA) was added to each well and incubated for 3 h in a CO2 incubator. The plate was incubated at 37 °C for three hours after 20 μl mg/mL 5 MTT (Sigma, USA) was added to each well. Subsequently, 100 μl of DMSO was added, the MTT solution was eliminated, and a micro-plate reader (BMG LabTech, Germany) (Abu-Serie et al. 2025) was used to measure the absorbance of each well at 570 nm. GraphPad Instat was used to find the CuONP dose values for the concentration that inhibits 50% of cell growth (IC50) and the concentration that is fully effective (EC100) at 50% and 100% cell survival, respectively.

### Statistical analysis

Microsoft Excel 2007 was the programme used to process the data. To compare the means, the collected data were statistically subjected to analysis of variance (ANOVA) as a factorial in complete block design (Gomez 1984) which was followed by Duncan's multiple range tests (Duncan 1955).

## Results

### Evaluation and characterization of the synthesized CuONPs

#### Ultraviolet–Vis spectral analysis

**Fig. 1:**
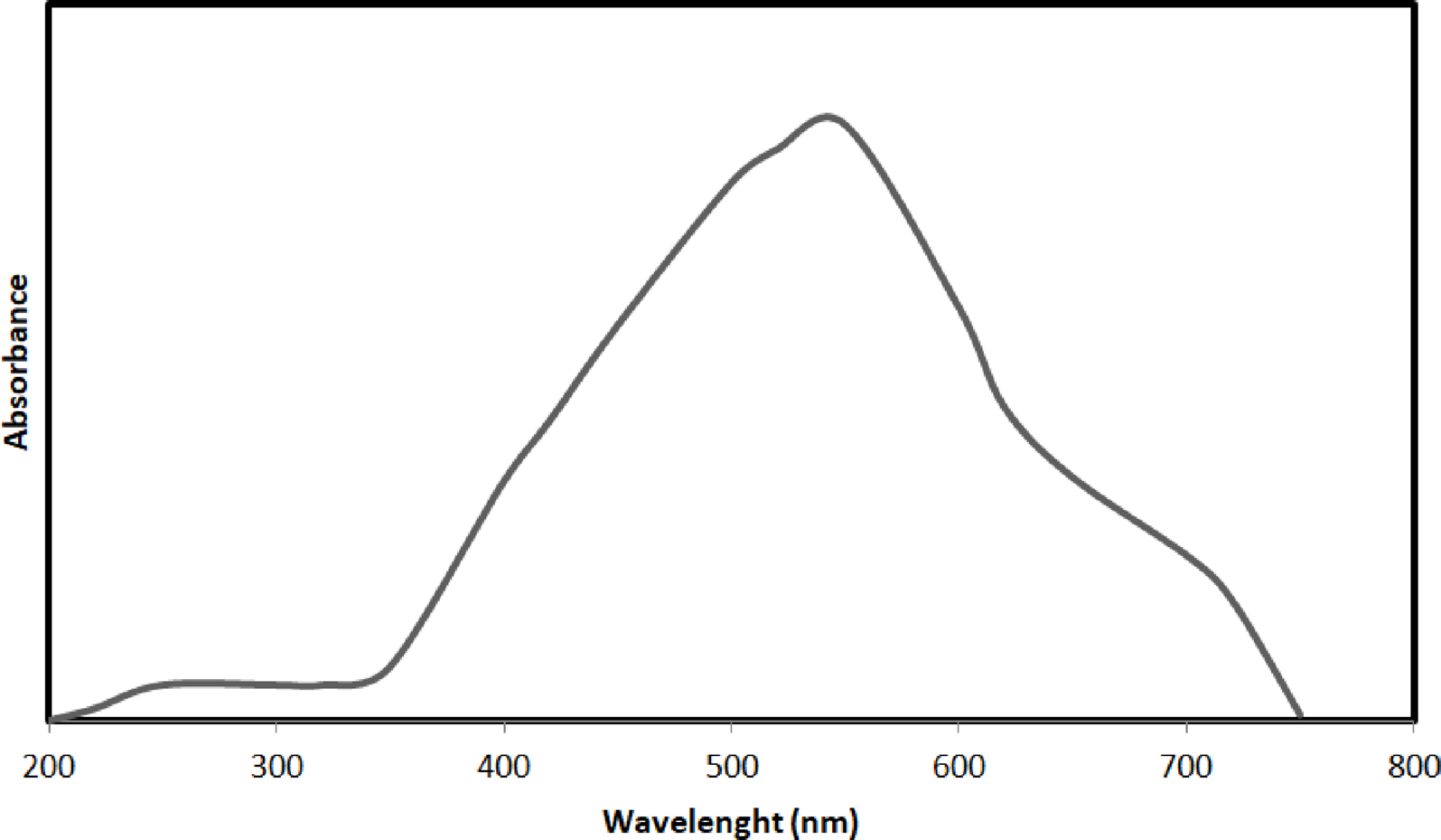
UV–Vis spectra of transformation CuONPs

The formation of copper nanoparticles was established with visual assessment. The UV–vis absorption spectra of copper nanoparticles are illustrated in Fig. 1. This absorbent-specific peak in the 550–590 nm range indicated a Surface Plasmon Resonance (SPR).

### Characterization of CuONPs

The synthesis of nanoparticles requires characterization, which is a crucial step in figuring out each copper nanoparticle's morphology, surface chemistry, surface area, and differences in nature. Copper nanoparticles are characterized in this study using FT-IR, XRD, EDX, Zeta potentials, SEM, and TEM techniques.

### X-ray diffraction analysis

**Fig. 2:**
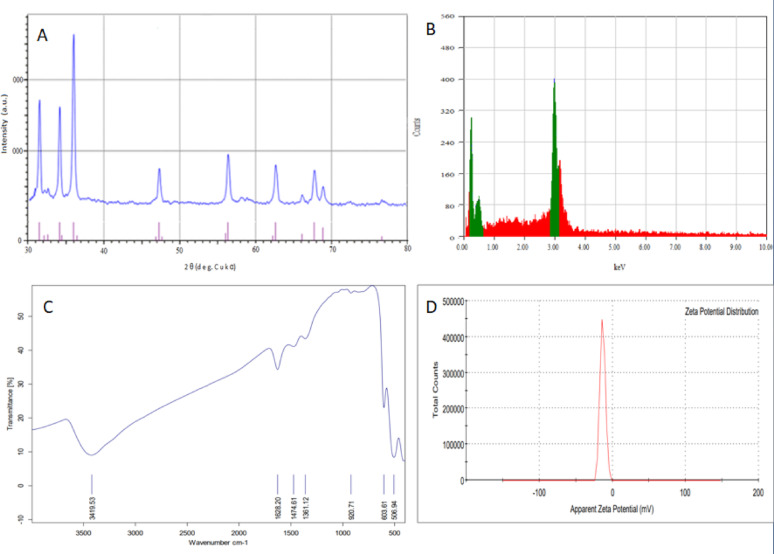
Characterizations of CuONPs; **A** XRD diffraction; **B** EDX spectrum image; **C** FT-IR- analysis; **D** Zeta potential distribution analysis

The chemical and structural constituents of the CuONPs sample were dedicated to X-ray diffraction. The representative XRD patterns of the CuO nanoparticles are present in Fig. 2A. In the XRD pattern, compared with the standard diffraction peaks from JCPDS card no. 80-1917, the peaks detected at 2θ values of 30–80° may be categorized as the typical monoclinic diffraction point CuO (a = 4⋅689 Å, c = 5⋅132 Å). The peak strength and widths indicated that the sample was extremely crystalline. Compared with the caliber diffraction patterns, no other characteristic peaks detected impurities, revealing that all the results were phase-pure.

### The energy dispersive X-ray and zeta potential distribution

Element analysis was carried out regarding EDX, an examination to assert the existence and quantification of the elemental constituent. Figure 2B of the EDX spectra revealed the existence of Cu and O, besides manifesting the values of the quantitative atom and weight percentage present in the CuONPs 27.7 Cu and 52.5% O. To obtain insight into the solidity of the synthesized CuONPs and determine the surface charge.

### FTIR- analysis

The FTIR spectra of CuONPs with the identical pattern of FT-IR 8400S (Shimadzu, Japan), are shown in Fig. 2C The surfactant 8400S was determined by comparing the two IR patterns in each sample. Peaks at 1419 cm are earmarks of the absorption of symmetrical stretching of the carboxyl group. A peak at 1682 cm⁻^1^, was recorded of C=O stretching vibration. And the peak at 1474 cm^−1^ may be allocated to the stretching modes 8 a, 8 b, and 19 as of the pyridyl group, respectively. Around 1635 cm^−1^ is accredited to the hydrogen bonds deployed from the hydroxide groups. As well, curve frequencies for –CH3 asymmetric and –CH3 symmetrical were recognized in 1452. The vibration at 1361 cm^−1^ is correlated with (C–H) and (C–H2) crooks. The band is marked at 920 cm^−1^ illustrating the existence of proteins. FT-IR analyses identified sulphate, a peak pointed out by vibration frequency at 603 cm^−1^. Finally, in the case of CuFeO_2_ NPs, we can observe a sharp peak at the absorption band at 506 cm-1 due to the band located at 555 cm^−1^ correlated to the stretching mode of CuO indicating the metal oxide nature of the NPs.

### Zeta potential analysis

The zeta potential analysis was carried out to observe the surface charges that CuONPs acquired. This can be done to ensure a clear prediction of the stability of the generated colloidal CuONPs. The zeta potential magnitude provides information about the colloids intrinsic stability. In this investigation, the CuONPs zeta potential was determined to be − 13.00 mV 2(D zeta).

The electrostatic repellent force between nanoparticles relies on the charge that occupies the surface of NPs. As a result, long-term stability results from the absence of nanoparticle aggregation in situations where the charge of the nanoparticles is negative, Fig. 2D.

### Scanning and transmission electron microscope

**Fig. 3:**
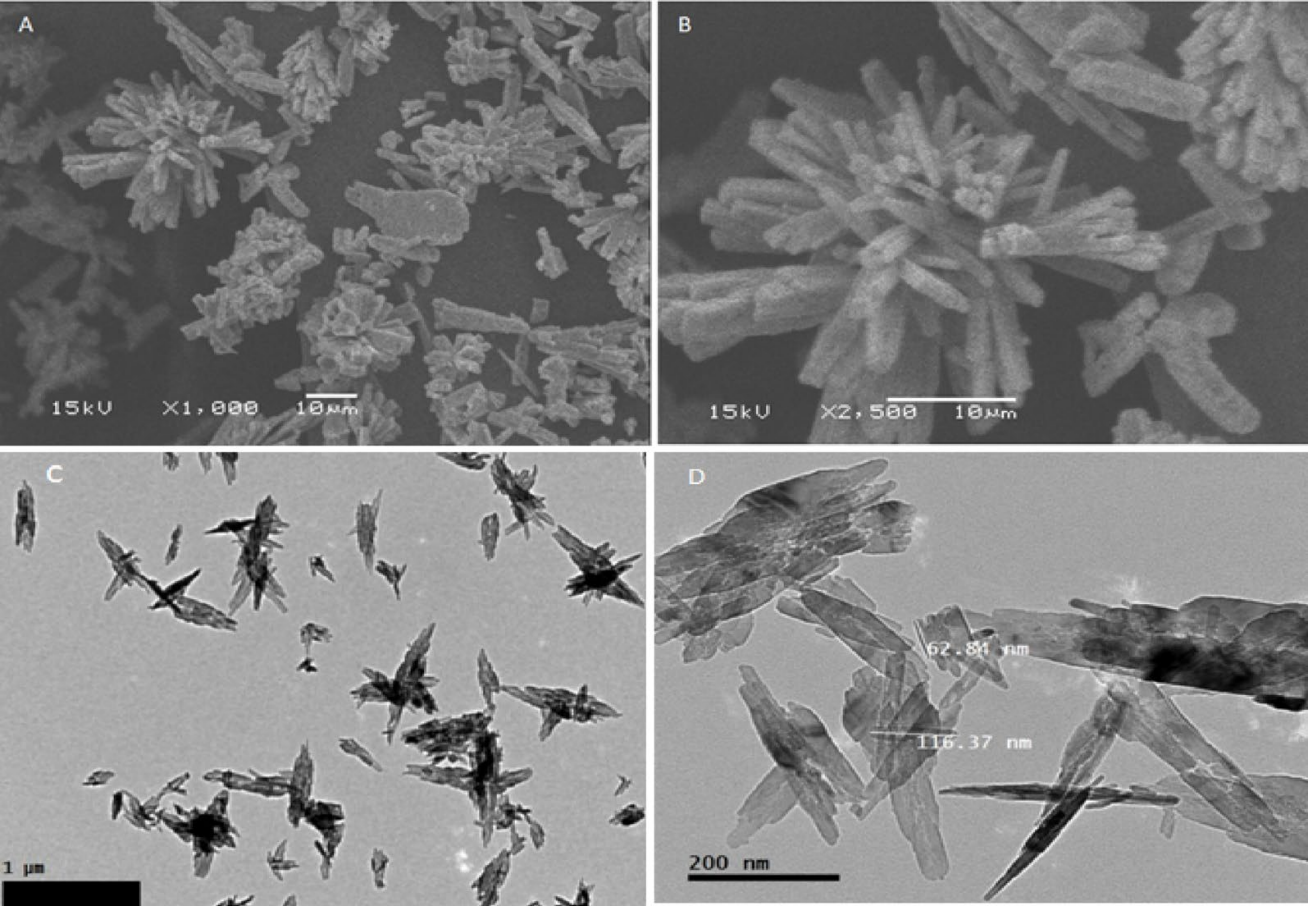
**A** and **B** photograph a high-magnification Scanning Electron Microscopy and **C** and **D** Transmission electron microscope of synthesis CuONPs

The construction of synthesized copper oxide nanoparticles revealed by SEM is illustrated in (Fig. 3A and B). This determines the distribution of the particles, the assimilation of particle sizes, and the surface characteristics of CuONPs. It mainly appears as a flower of agglomerated CuONPs and many aggregates containing synthesized nanoparticles. The transmission electron microscope (TEM) is the most commonly used tool for discovering nanoparticle samples' size, construction, morphology, scuttle, and biological and physical orientations. (Fig. 3C and D) illustrates ideal TEM images of CuONPs, which have a rectangular and pointed piece shape with many scattered nanoparticles of size about 62.8 to 116.37 nm with wide size dispersion.

### Meloidogyne incognita juvenile mortality and eggs hatchability in vitro assessment

**Fig. 4:**
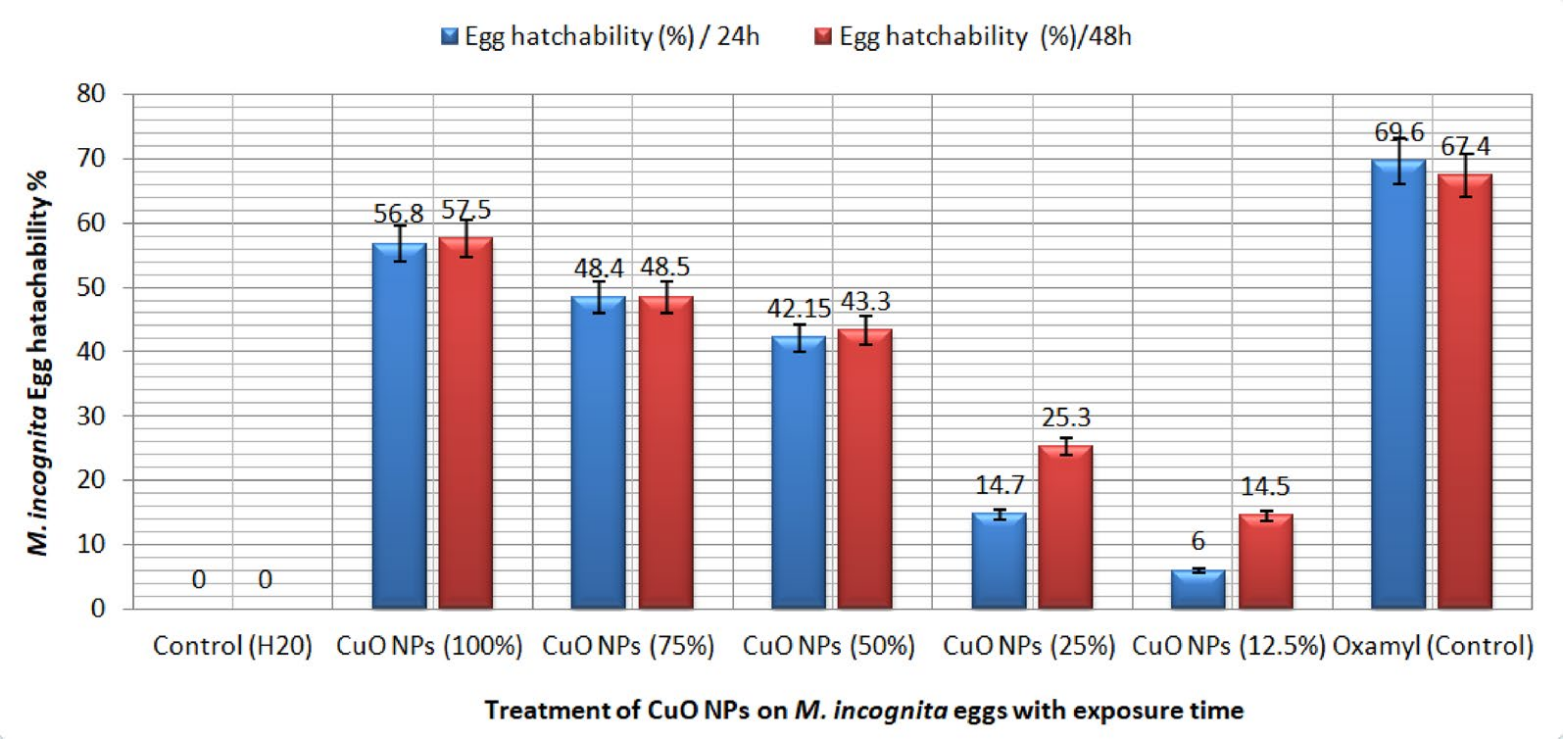
Impact of five CuONPs concentrations on the in vitro hatchability reduction of *M. incognita* eggs

The hatching percentage of *M. incognita* eggs (%) decreased significantly, and this decrease increased proportionally with increasing concentration from 12.5, 25, 50, and 75, to 100% at the time of exposure to CuONPs in vitro, (Fig. 4).

However, the percentage of eggs that hatched was decreased to 56.8, 48.4, 42.15, 14.7 and 6%, respectively, when *M. incognita* eggs were submerged in synthesis CuONPs with concentrations of 100, 75, 50, 25, and 12.5% respectively., after 24 h of exposure time; It also led to a decrease in the number of eggs by 57.5, 48.5, 43.3, 25.3, and 14.5%, with concentrations of 100, 75, 50, 25, and 12.5%, respectively after 48 h of exposure compared to the commercial pesticide (Oxamyl 24L), which reduced eggs to 69.6 and 67.4 after 24 and 48 h, respectively, when all treatments were compared with the untreated treatment (H_2_O).

**Fig. 5:**
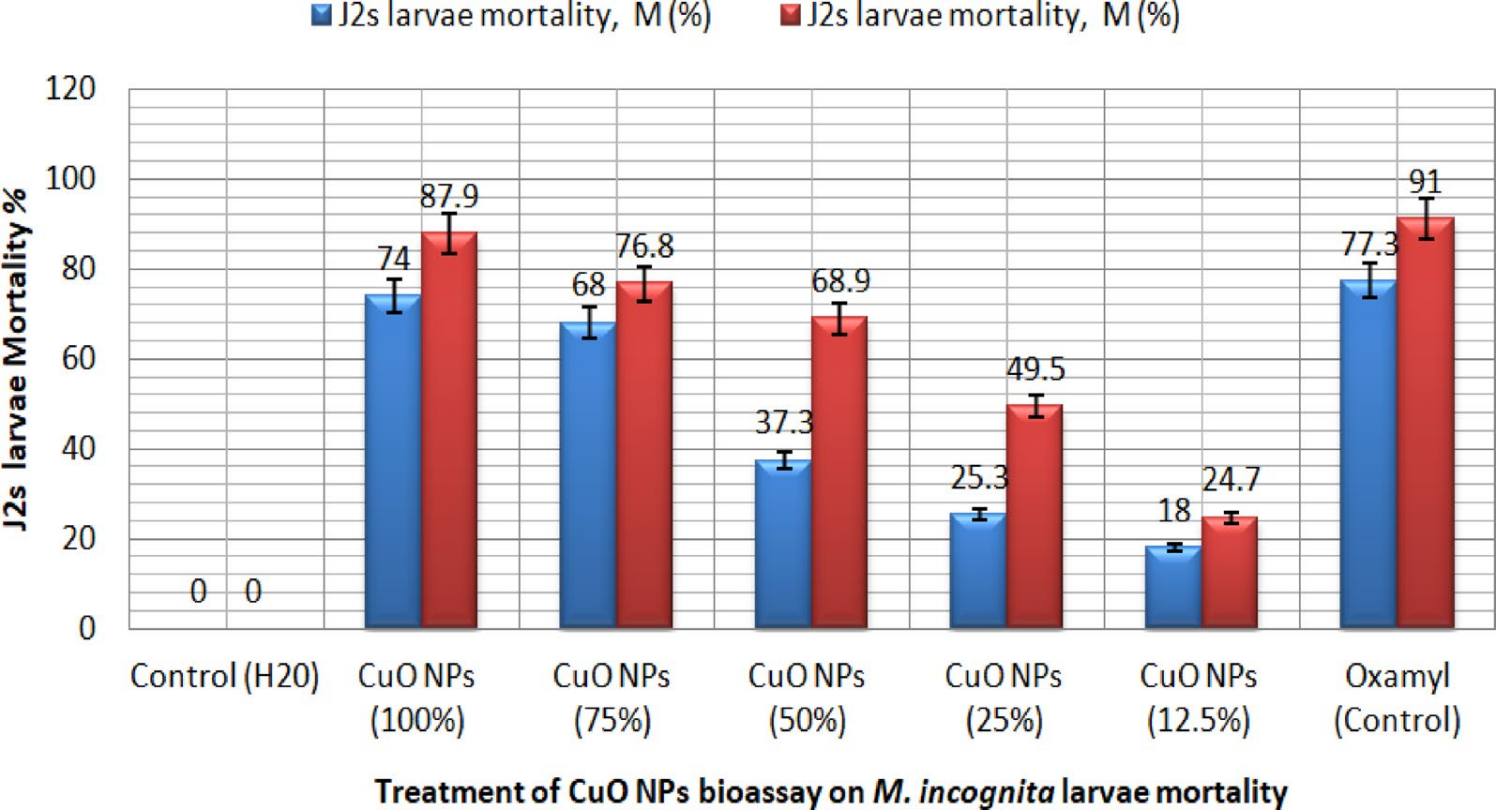
Impact of five CuONPs concentrations on the in vitro mortality rate of *M. incognita* J2s

In the case of examining the effect of synthesized CuONPs on second-stage juveniles of *M. incognita* (Fig. 5), similarly, the results of the highest CuONPs concentration provided the highest J2s mortality%, where CuONPs at a concentration of 100% led to juvenile mortality, followed by a concentration of 75%, with percentages of 74 and 68% J2s mortality rate after 24 h of exposure, respectively; likewise, were 87.9 and 76.8% mortality rates after 48 h of exposure, respectively; compared to oxamyl 24L, which resulted in 77.3 and 91% after 24 and 48 h of exposure, respectively. As well as the two concentrations (50 and 25%), they caused mortality of J2s *M. incognita* at a moderate effect of 37.3 and 25.3% after 24 h of exposure, respectively; they also led to mortality rates reaching 68.9 and 49.5. % after 48 h of exposure.

### Safety impact of CuONPs on the proliferation of human normal cells

**Fig. 6:**
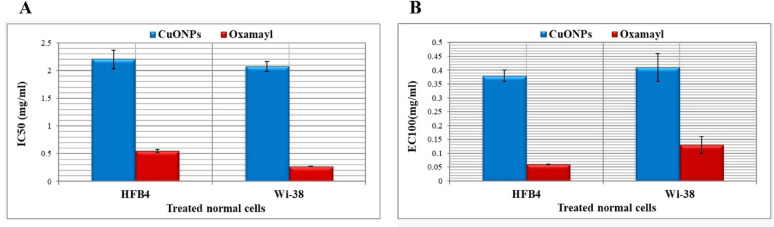
Cytotoxicity of CuO nanoparticles on both human normal cell lines (HFB4 and WI-38), as demonstrated in terms of (**A**) safe dose (EC100) and (**B**) dose at 50% cell viability (IC50)

As shown in Fig. 6A and B CuONPs had higher IC50 and EC100 values (≥ 2 and ≥ 0.15 mg/mL) than Oxamyl (≤ 1.1 and ≤ 0.13), indicating CuONPs were safer than this standard insecticide on the growth of human normal cell lines (WI-38 and HFB4, respectively).

### Efficacy of CuONPs of root-knot nematode parameters on pepper (plant roots and soil), open-field

**Fig. 7:**
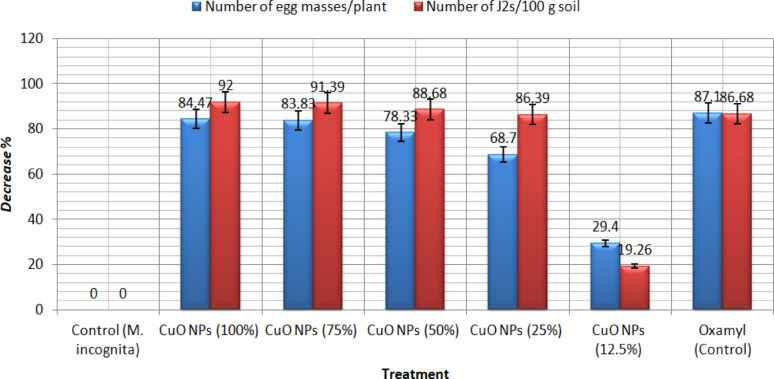
Effects of five CuONPs concentrations on the percentage decline in the number of egg masses/plant and J2s/100 g of *M. incognita* soil

The data in Fig. 7, illustrates the impact of synthesized CuONPs compared with oxamyl 24L on *M. incognita* gall numbers/root, egg masses/root of pepper planted in the open field, and the number of second juvenile stage/250 g soil after 60 days. Results show that CuONPs with a concentration of 100%, followed by 75 and 50%, reduce the nematode gall number and eggmasses over the control by (89.6–84.4%), (88.9–83.8%), and (82.9–87.3%) compared with the negative control (90.6–87%), respectively. Also, reduction of the numbers of J2s/ 250 g soil with a percentage of 92, 91.2, and 88.8% reduction compared with 86.6% by oxamyl 24L.

### Efficacy of synthesized CuONPs on root-knot nematode, M. incognita life cycle

**Fig. 8:**
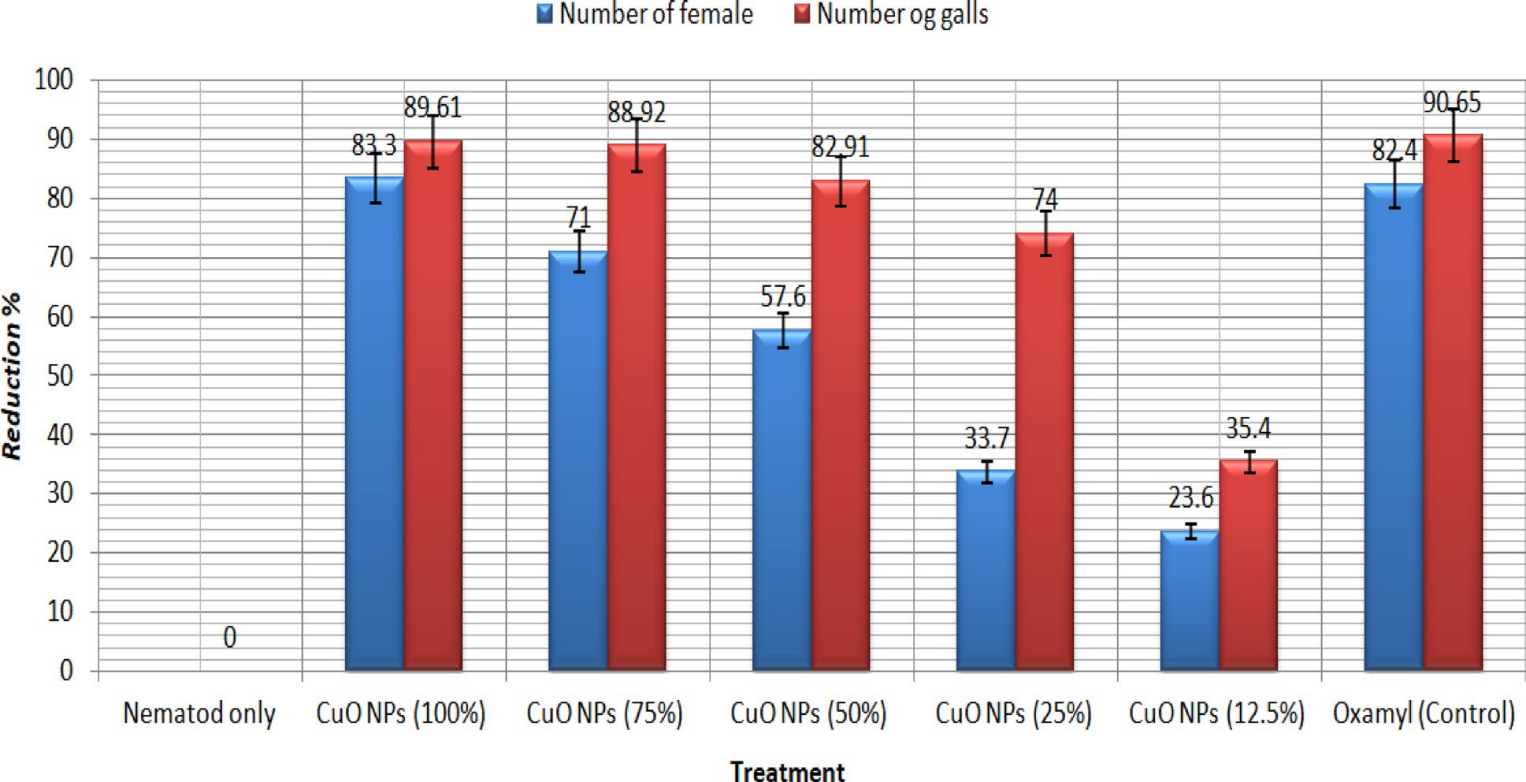
Effects of five CuONPs concentrations on life cycle of *M. incognita*

CuONPs were estimated in vivo in open field experiments with sweet pepper against the root-knot nematode *Meloidogyne incognita* which was naturally infested and clear dose response relationships were established based on the numbers of females and galls/g of root tissues. The reduction percentage of females/ pepper root tissues for CuONPs with concentration 100, 75, and 50 was examined and calculated (estimated) at 0.03, 0.05 and 0.2%/g of soil respectively. Similar percentages were found considering the reduction of galls/g of root tissues (Fig. 8).

### Assessment of the impact of CuONPs on the growth parameters of pepper plants in open field

**Fig. 9:**
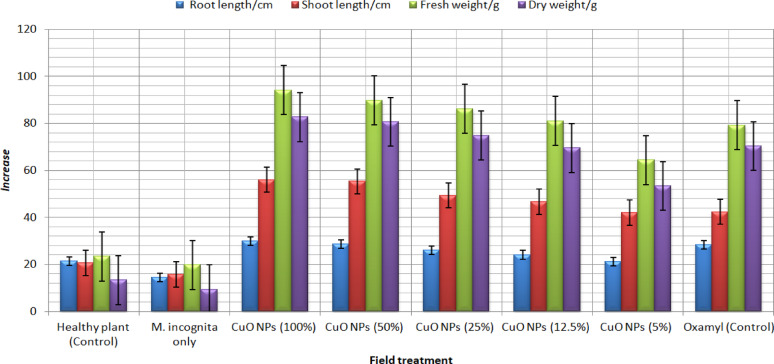
Effect of five different concentrations of CuONPs on percentage growth of pepper plant

To illuminate the mechanisms of synthesized CuONPs in plant growth responses, the pepper plants were grown and treated with CuONPs infested with *M. incognita* during open field conditions. The efficacy of CuONPs on the shoot and root growth parameters of pepper under open field conditions were explored in terms of a shoot system (length and fresh weight), and biomass (shoot dry weight), Fig. 9, displays the shoot height 55 days after seeding and treatment with 100, 75, 50, 25, and 12.5% CuONPs concentrations. The plants treated with these concentrations outperformed those treated with water or commercial nematicides. In particular, Fig. 9, show that CuONPs increased plant height (shoot and root) in centimeters when a concentration of 12.5% was reached. The plant height increased by 82.5 cm^2^, its dry weight increased by 35.9 g, and its fresh weight increased by 66 g when exposed to 25%, 50%, and 75% CuONPs, and 44, 77, and 88 g/plant mass, respectively. In contrast, the concentration of 100% CuONPs had the highest results in terms of positive effect on the plant, as it achieved 88 g for dry weight and 63 g for fresh weight compared to the control and pesticide treatment.

## Discussion

*Meloidogyne* spp., root-knot nematodes are hard to control and the agricultural regions need safe and effective nematicide in that command. Nanotechnology has offered a new perspective and approach to solving these agricultural problems; the performance of traditional pesticide formulations is often reformed through the development of nanotechnology (Khot et al. 2012). A plethora of physical, chemical, and biological techniques persist in progress and develop, directing to the production of nanoparticles from noble metals, which are used against the infestation of crops with nematodes (Bhau et al. 2016). In this context, we created copper nanoparticles chemically to investigate their ability to kill nematodes, and we thoroughly characterized the final product. The UV–visible spectroscopy in Fig. 1 gives a demonstration of the peak absorbance required for surface plasmon resonance at 580 nm, which is boosted by the peak at 631 nm stated earlier (Harrington et al. 2016) for the CuONPs SPR. As well, the peak absorbance for CuONPs was measured at 570 nm using both chemical and radiolytic cutting methods (Zhu et al. 2007). An X-ray diffraction confirmed the structure and chemical composition of the CuONPs sample. The typical XRD patterns of this sample are shown in (Fig. 2). The peaks in the XRD pattern match the standard peaks from JCPDS and indicate the unique distinctive diffractions of monoclinic phase CuO (a = 4.689 Å, c = 5.132 Å). According to the peak widths and intensities, the sample was highly crystalline. Unlike typical diffraction patterns, there are no extra peaks indicating impurities, which mean the product is pure (Zou et al. 2011). According to SEM images in (Fig. 3A and B), the flower-like CuO nanostructures are made up of numerous interconnected nanosheets that range in thickness from 60 to 80 nm and in width and length from a few micrometers. The synthesized CuO sample was subjected to EDX analysis using a field emission scanning electron microscope (FESEM). Quantitative analysis and the presence of Cu and O peaks are visible in the EDAX spectra showing that Cu and O have a 1:1 stoichiometric proportion. Consequently, it was clear that the sample is made up of a monoclinic phase of CuO that is pure and consistent with the pattern of XRD. The prepared CuO sample's morphology was examined as illustrated in (Fig. 2A–D) by the FESEM and TEM Fig. 3, with the lower magnification, showing that the structure of the obtained CuO is shaped like a flower. The micro-flower has a diameter of roughly 6 μm. Also, the synthesis method used can largely determine the characteristics of Cu nanoparticles, so it is interesting to find out what might occur during the formation of CuONPs. Consequentially, the mechanism of formation of the proposed NPs is a novelty. The shape-like flowers' formation mechanisms differed when various preparation techniques were employed. The needle-like, shape-like, and flower-like CuO nanostructures obtained by hydrolyzing Cu (OAc) 2 solutions without using a surfactant determined that the morphology of the CuO crystallites depends on the increasing rates of different crystal facets. The zeta potential value was − 13 mV, which revealed the solidity of the nanocolloid; the values were similar to the earlier reported values of − 14.9 mV (Arratiaa et al. 2019). CuONPs were synthesized and tested as nematicides in open-field and in *vitro* studies, and their cytotoxicity was assessed. Specifically, *M. incognita*, a species of root-knot nematode, was examined. In lab and field studies, copper nanoparticles at different concentrations were successful in decreasing egg hatchability Fig. 4, and killing of *M. incognita* larvae Fig. 5. In contrast, during open-field experiments, CuONPs increased the growth parameter of pepper plants. Copper salt has previously been stated to have nematicide activities when applied in incorporation with maleimide derivative (Eloh et al. 2016). Specifically, copper sulfate, which is frequently applied as a fungicide to eradicate fungi and cure copper-deficient soils, has shown an EC50 value of 280 μg/mL against root-knot nematodes *M. javanica* (Rahul et al. 2014). Additionally, using copper sulfate and maleic acid together decreased RKN disease in tomatoes by 51.72% in pot trails, reduced the formation of galls with melon, and decreased the population of nematode soil counts (Yeon et al. 2019).

CuONPs in this study showed that giant activity with higher ionic emission levels happens 24 h after exposure to a concentration, with a 19.8 μg/mL mortality rate. Considering that the nematicidal activity of copper nanoparticles against egg hatchability Fig. 4 has been determined after 48 h with a reduction percentage of 34%, the release of copper ions 48 h after dispersion may be partly responsible for the strong activity of CuONPs in water, (Gkanatsiou et al. 2019). While coppers nematicidal activity against *M. incognita* has been reported to be 126 ± 48 μg/mL (Ghareeb et al. 2022). The pepper plant in the open-field experiment was infected with *M. incognita* and distinct physiological. The results showed that CuONPs are strong nematicides against root-knot nematodes (*M. incognita*) of 50 and 100% concentrations. A similar study (Eloh et al. 2016) has examined the nematicidal effects of CuO nanoparticles against *Meloidogyne* species and reported that the copper salts combined with the maleimide derivative have nematicidal effects; the 200 ppm CuO nanoparticle concentration in the cowpea plants infected with nematodes causes a decrease in the nematode parameters. Also, CuO nanoparticles could be responsible for growth-enhancing and evolution of the plant (Baldwin and Bell 1981), while the reduction percentage of nematode infection in cowpea seedlings caused by the root dip method is directly related to nanoparticle absorption, the decrease in infection percentage refers to a decrease of J2s toward the root system and a shortage of root secretions in the existence of CuONPs.

Further research is needed to determine the mechanisms of action of copper nanoparticles on root-knot nematodes. It has been reported that heavy metals cause toxicity in *Caenorhabditis elegans* nematodes by rupturing of the nematode cell membrane, which results in a high mortality rate from the degradation of nematode cellular structure caused by nanoparticle treatment (De Oliveira et al. 2014). Furthermore, Cu + 2 and other heavy metal ions affect how neurons function because they lower cellular energy by changing the mitochondrial function, increasing stress through the generation of ROS, and starting the processes that lead to cell death (apoptosis, necrosis).

Accordingly, this study in Fig. 6 revealed that CuONPs are useful for improving the vegetative growth parameters of peppers. Specifically, there were noticeable differences in plant length by 99.54 and 55% and root length by 66 and 45% of CuONPs concentration at 50 and 100%, respectively. Furthermore, following CuONPs treatment, a higher ratio of plants increased in height and fresh, dry weight.

Additionally, the beneficial impact of CuONPs was noticeably greater than the effect of the commercial oxamyl treatment. It is commonly known that applying copper at lower concentrations promotes plant growth, enhances crops, and contributes outstandingly as a micronutrient (Karlsson et al. 2009). Copper is widely distributed in plant tissues and plays a crucial role as a micronutrient in regulating physiological and growth processes (Khan 2022).

Copper nanoparticles are used as antimicrobial agents (Ruparelia et al. 2008), in addition to medications to treat osteoporosis (Antonio-Pérez et al. 2023).

Additionally, pesticides contain significant amounts of nano-copper (Adisa et al. 2019; Parada et al. 2019). Human exhibition may be at risk due to this intersection with chain food, but direct medical intervention using nano-copper can deliver copper nanoparticles directly into the bloodstream, avoiding defense mechanisms through other conventional exposure routes like ingestion and inhalation. Although studies on the exposure of animal models to copper nanoparticles have been conducted (Song et al. 2015; Lee et al. 2016), comparatively little is known about the direct effects of copper nanoparticles on human cells and whether or not different particle sizes carry different risks. This size dependence has been noted for other particles as well, such as silver nanoparticles, another common antimicrobial metal nanoparticle (Kennedy et al. 2019). Further in vitro data are required to ascertain how the particles might change in biological matrices and whether certain nanoforms are more likely to cause cellular cytotoxicity before in vivo investigations can take place.

To determine whether using such particles as a pesticide against plant parasitic root-knot nematodes we assessed the toxicity of CuONPs on two cell lines (WI-38 and HBF4).

In evaluating CuONPs cytotoxic potential using a cell line as a test subject, the recorded EC100 values of CuONPs were 1.78 and 4.23 μg ml^−1^, for WI-38 and HBF4, in that order (Abu-Serie et al. 2025).

According to the current results (Fig. 6A, B), the cytotoxicity values (IC50 and EC100) of these nanoparticles were ≥ twofold higher than the standard insecticide. This declared that CuONPs exhibited a higher safety impact on the growth of both normal cell lines. It is in line with the current finding, a previous study reported that applying Cu nanoparticles to cells was non-toxic (Elbialy et al. 2023). Furthermore, another study concluded that the copper nanoparticles with chitosan showed low toxicity values against the normal cell line WI-38 (Adhikari et al. 2018). However, these copper nanoparticles with chitosan possessed powerful cytotoxic activities against cancer cell lines.

## Conclusions

In conclusion, we report the nematicidal effects of CuO nanoparticles that were further characterized using UV–vis, XRD, SEM, TEM, FT-IR, and Zeta potential spectroscopy techniques. As shown in this study, the graded concentrations of copper nanoparticles significantly enhanced and improved the growth parameters of pepper plants as well as suppressed of the nematode symptoms, which was determined to be most promising. Interestingly, the root-knot nematode recorded a maximum mortality and reduction at the higher concentrations (100%). The results clearly show that CuONPs can be used in watering plants to help move materials into their cells, which is a smart and useful way to boost plant growth and production while also protecting against harmful plant parasitic nematodes. These CuONPs could be helpful as safe and non-toxic nematicides or nanofertilizers, suggesting they might be a strong alternative to current pesticides.

## Data Availability

No datasets were generated or analysed during the current study.
